# Variance in population firing rate as a measure of slow time-scale correlation

**DOI:** 10.3389/fncom.2013.00176

**Published:** 2013-12-06

**Authors:** Adam C. Snyder, Michael J. Morais, Matthew A. Smith

**Affiliations:** ^1^Department of Ophthalmology, University of PittsburghPittsburgh, PA, USA; ^2^Center for the Neural Basis of Cognition, University of PittsburghPittsburgh, PA, USA; ^3^Department of Bioengineering, University of PittsburghPittsburgh, PA, USA; ^4^Fox Center for Vision Restoration, University of PittsburghPittsburgh, PA, USA

**Keywords:** Spike count correlation, electrophysiology, population coding, variance, noise

## Abstract

Correlated variability in the spiking responses of pairs of neurons, also known as spike count correlation, is a key indicator of functional connectivity and a critical factor in population coding. Underscoring the importance of correlation as a measure for cognitive neuroscience research is the observation that spike count correlations are not fixed, but are rather modulated by perceptual and cognitive context. Yet while this context fluctuates from moment to moment, correlation must be calculated over multiple trials. This property undermines its utility as a dependent measure for investigations of cognitive processes which fluctuate on a trial-to-trial basis, such as selective attention. A measure of functional connectivity that can be assayed on a moment-to-moment basis is needed to investigate the single-trial dynamics of populations of spiking neurons. Here, we introduce the measure of population variance in normalized firing rate for this goal. We show using mathematical analysis, computer simulations and *in vivo* data how population variance in normalized firing rate is inversely related to the latent correlation in the population, and how this measure can be used to reliably classify trials from different typical correlation conditions, even when firing rate is held constant. We discuss the potential advantages for using population variance in normalized firing rate as a dependent measure for both basic and applied neuroscience research.

## 1. Introduction

Cortical neurons emit variable spiking responses to repeated presentations of an identical stimulus. This variability has often been taken to represent the stochastic output of Poisson-like spiking generation, and therefore been subjected to averaging to estimate the “true” neuronal response to the stimulus. It is also clear, however, that measurement of the variability and its relationship to the mean response affords important insight into the mechanisms of neuronal coding. For instance, neuronal variability is reduced after stimulus onset in a bevy of cortical regions (Churchland et al., 2010), can reveal the neural mechanisms of decision-making (Churchland et al., 2011), and has been used to relate the activity of single neurons to behavior (for review see Nienborg et al., 2012). Our ability to understand variability, however, is constrained by single-neuron recordings. Noise in a neuron's responses to an identical stimulus might represent fundamental processes such as the diffusion of vesicles across a synapse (for review see Ribrault et al., 2011), but it also may reflect the influences of other, unrecorded neurons on the spiking of the neuron under study. A deeper understanding of the mechanisms of neural coding requires measurement of the relative contribution of these two factors, and in turn of how the interactions among neurons shape neural computations.

With the advent and recent popularity of recording devices capable of measuring the spiking responses of many single neurons simultaneously, it has become possible to measure the statistical interactions among neurons. The “noise” that occurs in response to repeated presentations of an identical stimulus can be also manifest as correlated trial-to-trial variability among groups of neurons. This indicates that the source of this variance is not entirely synaptic or channel noise, but can be at least partially attributed to common influences across the observed neurons. Spike count correlation (*r*_sc_, or “noise” correlation) is therefore indicative of a manner of functional connectivity between neurons. The structure of such correlated variability can reveal important features in the underlying neuronal circuits (Zohary et al., 1994; Shadlen and Newsome, 1998; Kohn and Smith, 2005; Smith and Kohn, 2008; Cohen and Maunsell, 2009; Mitchell et al., 2009; Ko et al., 2011; Smith and Sommer, 2013).

In addition to investigations of neuronal circuits, the magnitude and structure of correlated variability has profound consequences on the information processing capacity of neuronal populations. Consider, for example, a population of neurons all encoding the same stimulus with identical tuning curves. Although each neuron's response is somewhat unreliable, this could potentially be mitigated by pooling across the population to average out the noise. If the noise is correlated across neurons, however, then it will not average out completely and the encoding of the stimulus will be affected. In this simple example, correlated variability is potentially detrimental for stimulus encoding, although the full relationship depends on the structure of correlation with respect to neuronal tuning curves (Abbott and Dayan, 1999; Averbeck et al., 2006), and is different in a model built from populations of neurons with heterogeneous vs. homogenous tuning curves (Shamir and Sompolinsky, 2006; Ecker et al., 2011). The magnitude of spike count correlation is not fixed, however, and the brain appears to modulate correlation in a manner that is contextually favorable. For instance, correlations are altered in populations of neurons after the onset of a visual stimulus (Kohn and Smith, 2005; Smith and Kohn, 2008; Smith and Sommer, 2013), due to spatial attention (Cohen and Maunsell, 2009, 2011; Mitchell et al., 2009), perceptual learning (Gu et al., 2011), and association learning (Komiyama et al., 2010; Jeanne et al., 2013). Investigating the mechanisms responsible for neural correlations can therefore provide critical information about how the brain marshals processing resources in preparation for various perceptual and cognitive demands.

The use of correlated variability to provide a window into the state of a network does have a substantial limitation: it is only defined over multiple repeated trials. This precludes the use of correlation to investigate cognitive and perceptual processes on a single-trial, or moment-to-moment, basis. One measure that can be assayed from a neuronal population on a single-trial basis, however, is the population variance in firing rate measured across all the simultaneously recorded neurons. We proved that this latter measure is inversely related to the magnitude of correlation latent in the neuronal population (see Appendix), and reasoned that it can be used as a single-trial measure of functional connectivity. This inverse relationship between single-trial population variance and cross-trial pairwise correlation is rooted in the well-known Law of Total Variance (Weiss et al., 2005). We will demonstrate the applicability of this measure for identifying different correlations in realistic conditions using computer simulations and *in vivo* data. Our results can be taken as proof of concept that this method works in a simplified situation where firing rates are held constant and only correlation varies, although our mathematical derivation demonstrates that this relationship can be generalized to different firing rates. Thus, taking population variance into account will necessarily improve on using firing rate alone to estimate, on a single trial, cognitive states where correlation is a known variant.

## 2. Computer simulations

### 2.1. Materials and methods

#### Generation of simulated datasets

We investigated the ability by which population variance in normalized firing rate could discriminate between single trials drawn from low- and high-correlation regimes in simulated datasets. Specifically, we tested the sensitivity of classification to changes in population size and correlation difference between conditions. Simulated data were generated as thousands of trials of populations of correlated Poisson spike counts using the Dichotomized Gaussian (DG) algorithm created by Macke and colleagues (2009; http://www.bethgelab.org/software/mvd) and recently used by a number of studies to simulate population responses of correlated neurons (Savin et al., 2010; Xue et al., 2010; Long and Carmena, 2011; Schaub and Schultz, 2012). A DG algorithm uses a multivariate normal distribution that is thresholded onto 0 or 1 to construct a multivariate Poisson distribution, which produces a known and invertible mapping of the input statistics. This algorithm finds the inverse mapping for specified output statistics and samples accordingly from the corresponding normal distribution.

Populations of 120 neurons were simulated over 1000 trials for eight spike count correlation magnitudes evenly spaced between *r*_sc_ = 0.05 and 0.40. As stated above, inputs to the algorithm included a mean firing rate vector and a full covariance matrix. The firing rate distributions of all simulated populations were matched with respect to the means and standard deviations, which were set equal to the overall statistics of the *in vivo* data (see following section). As a result, simulated datasets differed from each other only with respect to their latent correlations. For each simulated dataset, all of the pairs of neurons had the same specified correlation coefficient; given the scale of our simulations (*N* = 120), this was necessary to ensure the algorithm reliably converged on a solution. Note that since correlation is not defined on a single-trial level, we use the term “latent correlation” throughout this report to indicate the correlation of the full dataset from which the single trial was drawn.

#### Normalization and classification procedures

**Figure 1:**
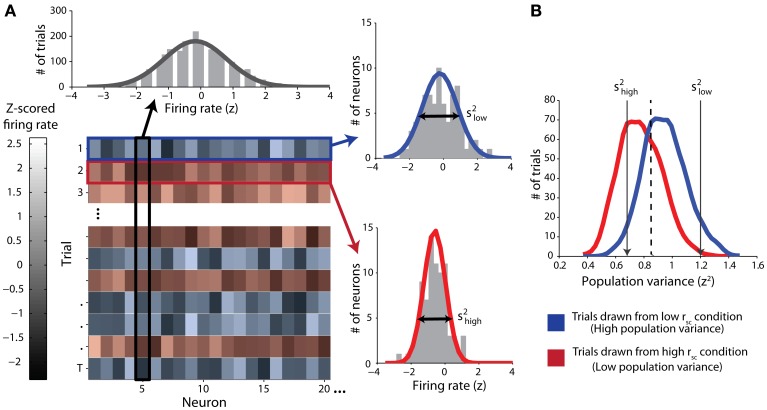
**Schematic of classification procedure using example simulated data**. Throughout the figure, red color represents data from the high-correlation condition and blue color represents data from the low-correlation condition. **(A)** Top: *z*-scored firing-rate distribution for one example simulated neuron. Trials from both correlation conditions are included. The solid line represents a Gaussian fit. Lower left: Matrix of data from all neurons (columns) and trials (rows). The lightness of the monochrome shading represents the normalized firing rate for the given neuron on the given trial. Right: example distributions of normalized firing-rates on single trials (top right: low correlation trial; bottom right: high-correlation trial). The variance of each distribution (*s*^2^) is the single-trial population variance. **(B)** Distributions of population variances for high and low correlation conditions for the simulated data illustrated in **(A)**. The arrows indicate the specific variances of the two single-trial distributions of normalized firing rates in **(A)**. Note that overall, the population variance for the high correlation condition (red) is lesser than the population variance for the low correlation condition (blue). We classified single trials by using a sliding threshold (e.g., the dashed line). Values above this threshold were classified as low correlation.

In classifying single trials, we sought to measure the ability of an ideal observer to discriminate between trials drawn from either correlation condition based only on the population variance statistics. We employed classical receiver operating characteristic (ROC) analysis to classify single trials drawn from pairs of simulated datasets, each corresponding to a particular correlation condition. First, and in line with our mathematical proof (see Appendix), we normalized the firing rates for each neuron by z-scoring across responses for all trials of all conditions for that neuron (Figure 1A, top). That is, all trials of both the low- and high-correlation conditions were used in the same normalization in an effort to capture the full range of each neuron's activity. After normalization of firing rate, we calculated the population variance (across neurons) of the z-scored responses for each single trial (Figure 1A, right). Trials with a low latent correlation tend to produce high population variance (blue), and trials with a high latent correlation tend to produce low population variance (red). Classification efficacy of population variance was determined using a sliding linear classifier with which we constructed ROC curves (Figure 1B). In accordance with our rationale, we classified trials with population variance above the classifier threshold (high variance trials) to be from the low correlation condition, and trials with variance below the classifier threshold (low variance) to be from the high correlation condition. Each point on the ROC curve represents the true positive rate of this rule against the false positive rate for a particular classifier threshold value. We also performed the identical classification procedure using mean normalized firing rate as a criterion to ensure no confounding firing rate effects. For both variance and firing rate analyses, classification ability was further quantified from ROC curves by integration to yield area-under-curve (AUC) measurements.

#### Classification of simulated datasets

We constructed pairs of datasets that varied on two parameters of interest: population size and difference in average pairwise correlation between conditions (Δ*r*_sc_). To vary population size while holding correlation difference constant, we started with a single pair of datasets with a correlation difference fixed at a value of 0.05 (*r*_sc_ = 0.05 vs. *r*_sc_ = 0.10), approximately equal to that which we observed *in vivo*. We tested this same value for Δ*r*_sc_ for a range of baseline correlation values and did not observe a systematic difference in the results. We then randomly subsampled this pair of datasets to build populations ranging in size from 10 to 120 neurons. To vary Δ*r*_sc_ while holding population size constant, we fixed one dataset at *r*_sc_ = 0.05 and paired it with datasets ranging from *r*_sc_ = 0.05–0.4, such that the pairs produced differences in correlation ranging from Δ*r*_sc_ = 0–0.35. We subsampled neurons from these datasets to match population sizes to the average population sizes reflected in our *in vivo* data, approximately 80 neurons. We classified all single trials from these pairs of datasets by both variance in normalized firing rate and mean normalized firing rate using the ROC methods given above. To reduce sampling error, AUC measures and ROC curves were constructed by averaging over 40 random subsamples.

### 2.2. Results

**Figure 2:**
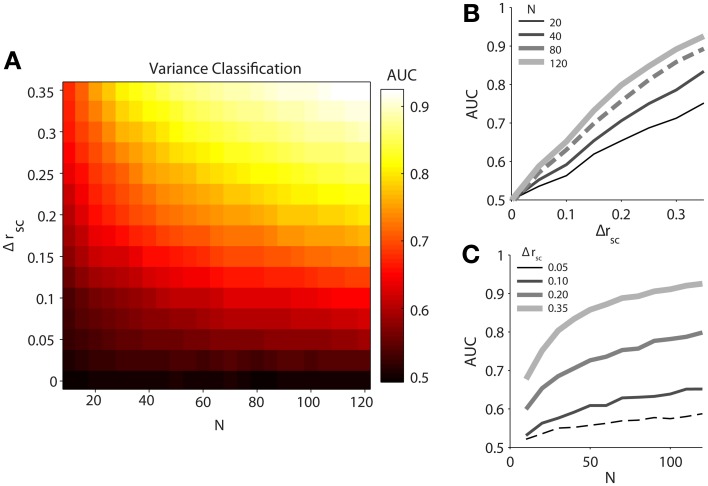
**Classification performance of population variance in normalized firing rate for simulated data**. We produced pairs of data sets for each classification condition by varying spike count correlation only. **(A)** Variance classification ability as a function of population size (*N*) and difference in spike count correlation (Δ*r*_sc_). Data sets were generated with the same number of neurons (*N* = 120), with one set having a spike count correlation of *r*_sc_ = 0.05 and the other having a value of *r*_sc_ = 0.05 + Δ*r*_sc_. **(B)** Classification ability of variance measure as a function of difference in noise correlation between conditions (Δ*r*_sc_) with population size fixed at *N* = 20, 40, 80, or 120 neurons. **(C)** Classification ability of variance measure as a function of population size (*N*) with a difference in noise correlation fixed at Δ*r*_sc_ = 0.05, 0.10, 0.20, or 0.35.

Our simulated data demonstrated that classification ability, measured by the area under the ROC curves (AUC), increases with both difference in latent correlation (Δ*r*_sc_) between and population size (*N*) of pairs of datasets (Figure 2A). It should be noted that classification using mean normalized firing rate was completely random (AUC ≈ 0.5) for any Δ*r*_sc_ or *N*, reflecting the identical firing rate terms we fed into the model. We fit the relationship between AUC and Δ*r*_sc_, holding the number of neurons fixed at 80 (Figure 2B, dashed line), with a first order rational function in the ordinary least squares sense. The fitted equation was $AUC=\frac{1.9\Delta r_{\text{sc}}+0.42}{\Delta r_{\text{sc}}+0.86}$ (*R*^2^ = 0.9992). As we understood that latent correlation in a population of neurons was related to the single-trial variance across the population, it followed directly that increasing the difference in latent correlation between two populations increased our ability to discriminate between them with the population variance measure. Even though the relationship appeared linear on the interval over which we measured it, we fit a rational function with the knowledge that AUC has an intrinsic maximum of 1.

We likewise fit the relationship between AUC and population size (*N*) with a first-order rational function, holding Δ*r*_sc_ fixed at 0.05 (Figure 2C, dashed line). The resulting fit was $AUC=\frac{0.62N+28.4}{N+56}$ (*R*^2^ = 0.9819). Unlike the latent correlation parameter (Δ*r*_sc_), the rational shape of the curve was evident on the interval over which we investigated it. Notably, after the population exceeded 70 neurons, a plausible number of neurons to be found on a single 96-electrode array, classification ability only increased slowly. This indicates that a sample size of roughly 70 neurons might serve as a target for researchers interested in using population variance as a proxy for latent correlation, as sample sizes below this level (e.g., as might be obtained with a linear electrode array or a tetrode) would provide a less reliable estimate of the true population variance around the recording site.

Despite the fact that single-trial variance in normalized population firing rate is a noisy approximation for latent correlation, we were able to classify trials into two correlation conditions with better-than-chance performance even for the smallest non-zero differences in correlation tested and with the smallest number of simulated neurons tested (*N* = 20). However, classification performance improved rapidly as the number of neurons sampled increased to around *N* = 70, at which point performance increases tapered off substantially. Across all sample sizes tested, classification ability improved roughly linearly with the difference in latent correlation for the range of values that we tested, which were chosen to be physiologically plausible.

## 3. *In vivo* data

### 3.1. Materials and methods

#### Subjects and surgical preparation

We recorded neuronal activity from two adult male Rhesus macaques (*Macaca mulatta*). Surgeries were performed in aseptic conditions and under isofluorane-induced anesthesia. Each animal was implanted with a head fixation post and a “Utah” microelectrode array (Blackrock Microsystems, Salt Lake City, UT) in visual area V4 of the right hemisphere. Details of array implantation in V4 have been reported previously (Smith and Sommer, 2013). Each microelectrode array consisted of a 10 × 10 grid of silicon electrodes (each 1 mm in length) spaced 400 μm apart. All procedures were approved by the Institutional Animal Care and Use Committee of the University of Pittsburgh and were in compliance with the guidelines set forth in the National Institutes of Health's *Guide for the Care and Use of Laboratory Animals*.

#### Data collection

Signals from each microelectrode in the array were amplified and band-pass filtered (250–7500 Hz) by a Ripple (Salt Lake City, UT) data acquisition system. For each electrode in the array, waveform segments that exceeded a threshold (periodically adjusted using a multiple of the RMS noise on each channel) were digitized (30 kHz) and stored for offline analysis and sorting. Waveform segments were sorted with an automated algorithm that clustered similarly shaped waveforms using a competitive mixture decomposition method (Shoham et al., 2003). We manually refined the output of this algorithm for each electrode with custom time-amplitude window discrimination software (“Spikesort”; Kelly et al., 2007; http://smithlab.net/spikesort.html; written in MATLAB; MathWorks, Natick, MA), taking into account the shape of the waveform and the distribution of interspike intervals. Data from each session were sorted separately. Following the offline sorting procedure, we computed the signal-to-noise ratio (SNR) of each candidate unit as the ratio of the average waveform amplitude to the SD of the waveform noise (Nordhausen et al., 1996; Suner et al., 2005; Kelly et al., 2007). Candidates that fell below an SNR of 2.0 were discarded.

#### Task

To present visual stimuli we used custom software written in MATLAB using the Psychophysics Toolbox extensions (Brainard, 1997; Kleiner et al., 2007). All stimuli were displayed on a CRT monitor with a resolution of 1024 × 768 pixels and a temporal refresh of 100 Hz viewed at a distance of 36 cm. We used lookup tables to correct for non-linearities in the relation between input voltage and phosphor luminance in the monitor. The mean luminance of the display was 39 cd/m^2^. All stimuli were presented in a circular aperture surrounded by a gray field of average luminance. For each animal, we mapped the spatial receptive fields of each channel by presenting small, drifting sinusoidal gratings at a range of spatial positions. We centered our stimuli on the aggregate receptive field of the recorded units. We also measured the spatial- and frequency-tuning of the recorded neurons for each animal by presenting drifting gratings at a range of spatial and temporal frequencies. For the experiment, we used the spatial and temporal frequency combination that maximally excited the recorded neurons.

The animals completed a simple saccade task. We monitored the animals gaze positions with an Eyelink 2000 infrared tracking system (SR Research, Mississauga, ON). Animals were required to maintain fixation within 1.2° of a 0.15° blue dot for 2 s. During this period, a drifting sinusoidal grating was presented at the aggregate receptive field area for the recorded neurons. For the analyses described below, we used the spike counts over the interval from 0.5 to 1.5 s following stimulus onset. After the 2 s fixation interval had elapsed, the fixation point was extinguished and a new dot was presented 8° away from the original fixation point in a random direction. The animals received a liquid reward for making a saccade to the new location. We presented gratings at two orientations (horizontal and vertical) in a block-randomized fashion to reduce the effects of stimulus adaptation.

#### Surrogate data construction

We predicted that population variance alone could be used to classify single trials originating from conditions of different latent spike count correlations. In practice, however, firing rate and variance are often conflated due to the quasi-Poisson nature of neuronal spiking activity. Nevertheless, our model predicts that population variance would be inversely related to latent correlation even if firing rate is held constant. To test our prediction, it was therefore necessary to obtain datasets that differed with respect to correlation but did not differ with respect to firing rate. For this purpose, we leveraged a known property of spike count correlations in visual cortex; namely, that correlation between a pair of neurons decreases as the distance between those neurons increases (Smith and Kohn, 2008; Smith and Sommer, 2013). Accordingly, we derived variance estimates for two conceptually different subpopulations of our full sample of neurons: 1) neurons located relatively near each other, and 2) neurons located relatively far from each other. Specifically, the “far” set was predicted to have lower correlation than the “near” set.

**Figure 3:**
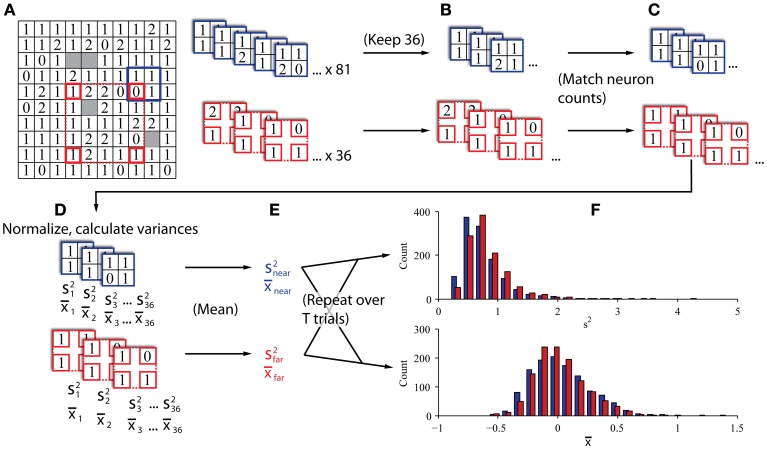
***In vivo* data sampling procedure. (A)** Single datasets preliminarily sampled by sliding near (blue) and far (red) kernels across the array to gather all candidate four-channel groupings. The 10 × 10 grid represents the configuration of the microelectrode array. The number in each box indicates the number of neurons recorded on the corresponding channel in a representative dataset from Monkey Bo. **(B)** If the two kernels produced different numbers of valid groupings (i.e., at least two channels with neurons), the larger set was subsampled to match the smaller set in the number of groupings, keeping only the groupings with the most neurons. **(C)** Neuron counts were reduced to match across kernels. When individual channels had more than one neuron, we selected the one with the highest SNR and discarded the others to exclude same-electrode pairs. **(D)** Neurons were normalized according to the full history of their recorded trials and the variance and mean were calculated across all normalized samples (*s*^2^ and *x*, respectively). **(E)** Variance and mean estimates were averaged within kernels to obtain an estimator of population variance and mean firing rate in populations of nearby and distant groups of neurons. **(F)** This procedure was repeated for all trials from each session to construct histograms of population variance and mean firing rate for eventual classification.

Our procedure is illustrated in Figures 3A–C. To create the “near” and “far” datasets we repeatedly drew subsamples from each trial in a session in groupings of four channels that were either 0.4–0.57 mm apart (“near”; blue elements in Figure 3) or 1.6–2.26 mm apart (“far”; red elements in Figure 3). Using the “near” dataset as an example, we moved the blue square to a position on the array and drew a sample of neurons at that position. The sample was discarded if neurons were not present on at least 2 channels. From that sample, we calculated the population variance and stored that value. We repeated this procedure for all possible positions of the blue and red squares (81 for “near” and 36 for “far”). We then averaged all the observed population variances to obtain a single value of population variance for the trial at each of the two distances, and repeated this procedure for every trial. This method was akin to a bootstrap resampling approach to estimate the variance. That is, we repeatedly sampled from the total population of all recorded neurons that were a particular distance from each other to derive a sampling distribution of variances, and used the mean of that bootstrapped sampling distribution as our estimate for the variance of the population of cells located at the given distance from each other. We tested different electrode configurations for the “near” and “far” conditions and found that the results were qualitatively robust for a wide range of configurations. In addition to calculating an estimate of variance for each condition, we also calculated the average spike count correlation for each condition, which allowed us to validate whether the two sets did indeed exhibit different magnitudes of correlation. We calculated the average firing rate for each condition to confirm that it was matched. Finally, we classified trials from the two conditions using a procedure identical to that described for the computer simulations above. All of the trials collected were used for the classification analysis (mean of 837 trials per session).

### 3.2. Results

**Figure 4:**
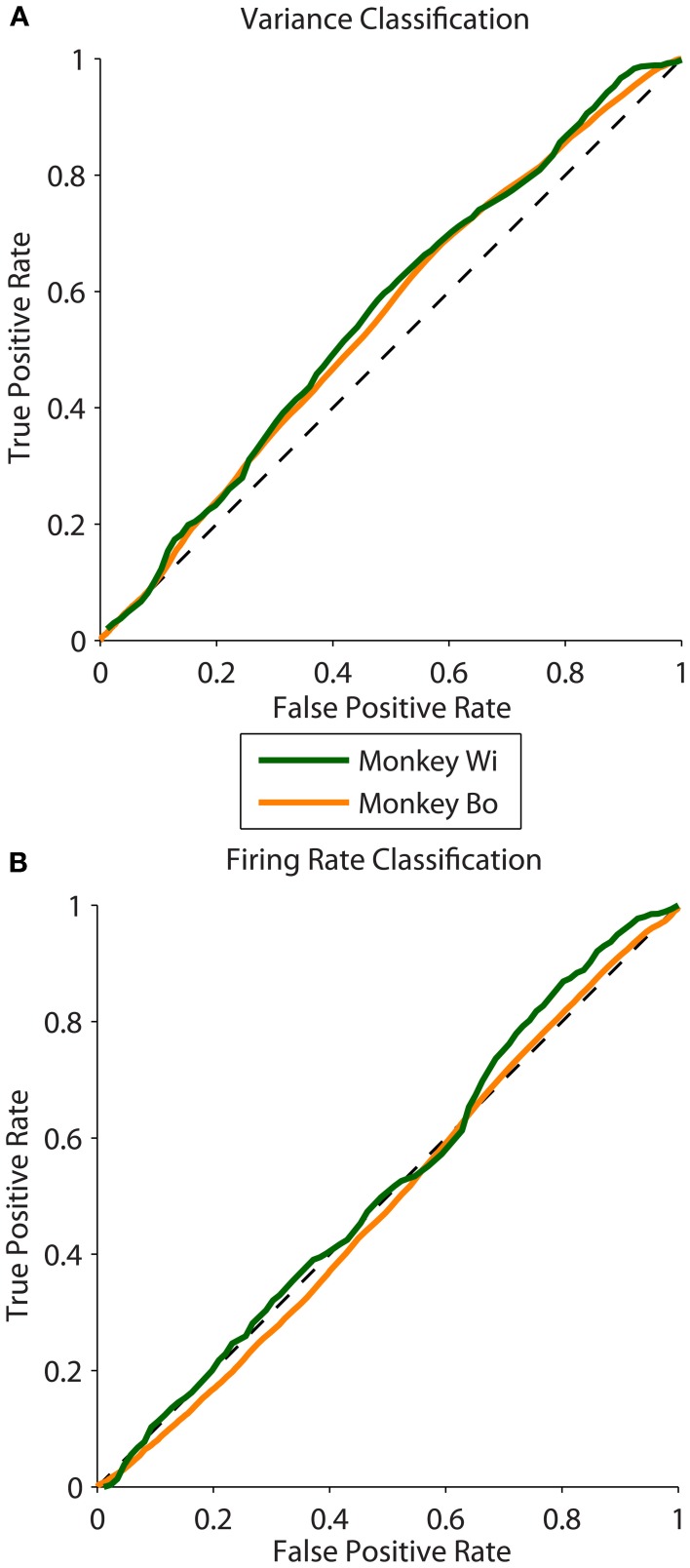
**ROC curves measuring classification ability across two animals. (A)** Sample ROC curves using population variance in normalized firing rate as a classifier. A single example dataset is plotted for each animal (corresponding to the datasets plotted with filled symbols in Figure 5). **(B)** Sample ROC curves using mean normalized firing rate for the same datasets as in panel **(A)**.

We analyzed 25 days of data from two monkeys, with 10 days for monkey Bo and 15 days for monkey Wi (typically 500–1300 trials per day). We found that the magnitude of the difference in latent correlation (Δ*r*_sc_) from near and far populations varied substantially; however, for all but 1 day, the difference we obtained was greater than zero. This non-zero difference in correlation between near and far conditions was reliably classified using the population variance measure, while mean firing rate, by construction, performed with random classification. Sample ROC curves from one example dataset of each monkey with robust differences in correlation are shown in Figure 4. These curves correspond to datasets with Δ*r*_sc_ values of 0.0476 and 0.0467 for monkeys Bo and Wi, respectively.

**Figure 5:**
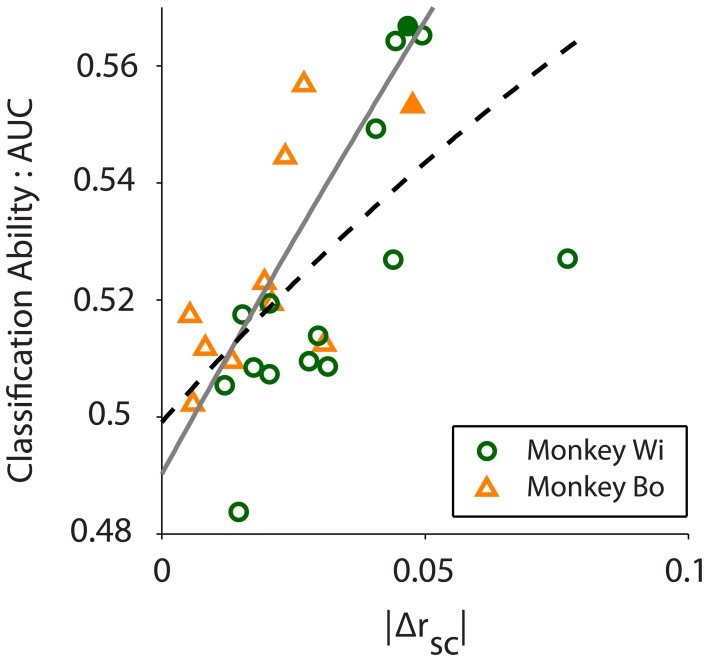
**Classification ability, measured by the area under the ROC curve (AUC), increases with absolute difference in correlation between conditions (|Δ*r*_sc_|; *r* = 0.610, *p* = 0.001)**. Scatter plot shows 10 and 15 days of *in vivo* recordings across two monkeys, Wi (green circles) and Bo (orange triangles), respectively. The black dashed line is a rational fit to the data in the least absolute residual sense (*R*^2^ = 0.347), to mitigate the effect of the outlier from Monkey Wi data. The gray solid line is a rational fit to the simulated data (see Figure 2B, *N* = 80 trace) in the least squares sense (*R*^2^ = 0.999). The filled symbols represent the example data sets presented in Figure 4.

As Figure 5 illustrates, the average difference in latent correlation measured with the near-far sampling method was in line with our expectation based on previously published V4 data (Smith and Sommer, 2013) at distances of 0.5 mm for near pairs and 1.5 mm for far pairs. For monkeys Bo and Wi, the average difference in correlation between the near and far conditions was Δ*r*_sc_ = 0.0183 and 0.0310, respectively. Despite these relatively small differences in *r*_sc_, we found that our algorithm was able to classify the near and far conditions based on population variance using the same procedures outlined above for simulated data.

We quantified classification performance as the area under the ROC curve. Classification using population variance was significantly better than chance (i.e., AUC > 0.5, right-tailed *t*-test, Bo: *p* = 0.0362, Wi: *p* = 0.0061). In contrast, classification was not better than chance when based on mean population firing rate (*t*-test, Bo: *p* = 0.4676, Wi: *p* = 0.4436). This was expected because our resampling procedure was blind to firing rate, and there is no principled reason why the near or far pairs from the same array would differ in firing rate. In our simulations we found that classification performance was related to the magnitude of the *r*_sc_ difference—a larger difference in *r*_sc_ made for better classification. In the *in vivo* data we also found this to be the case. Classification ability was related to the size of the difference in latent correlation measured for the datasets (Pearson correlation: *r* = 0.6100, *p* = 0.0012, Figure 5). We observed a substantially outlying data point from Monkey Wi (Figure 5, with a large Δ*r*_sc_ of 0.077 and an incommensurately low AUC of 0.527); to allay the influence of outlying data, we fit a rational function to the data using a least absolute residual robust fit (black dashed line). The resulting fit was $AUC=\frac{0.97\Delta r_{\text{sc}}+0.18}{\Delta r_{\text{sc}}+0.36}$ (*R*^2^ = 0.3472). For comparison with the simulation results, the first-order rational fit to the simulated data is also plotted (solid gray line). Although our *in vivo* data lie only in a restricted range of the simulation results, they agree well with the expected classification performance in that range.

## 4. Discussion

In this report, we showed mathematically that the single-trial population variance in normalized firing rate is complementary to the latent correlation in a population of neurons (see Appendix), and then tested this relationship in both simulated data and *in vivo* data. We found that population variance can perform moderately well at classifying data arising from different latent correlation conditions, even when the difference in correlation between those conditions is quite small, using physiologically plausible values of *r*_sc_ and realistically attainable numbers of neurons and trials. Using the simulations as a guide, we examined the relationship between population variance and *r*_sc_ *in vivo* and found a close match in classification performance.

Noise in neuronal responses can be viewed fundamentally in two ways, either as a nuisance to be averaged out over repeated trials or as signal that we have yet to uncover. Noise in neuronal responses has been related to neuronal computation and connectivity (Shadlen and Newsome, 1998), motor preparation (Churchland et al., 2006), decision-making (Churchland et al., 2011), and the limits of perception (Parker and Newsome, 1998). From the perspective of single-neuron recordings, it is difficult to separate true noise from meaningful functional interactions among neurons. A fundamental goal of neuroscience is to understand how neurons interact to encode and process information. This goal requires the observation of multiple neurons simultaneously so their interactions can be measured, and the noise in a neuronal population becomes a window into those functional interactions. As multielectrode recording technology continues to advance, it has become possible to record from dozens of neurons at a time, and these numbers are almost certain to increase. Making use of simultaneous recordings from large neuronal populations requires a framework of statistical understanding of neuronal interactions that can be built and tested using current recording methods.

Spiking activity in pairs of cortical neurons is correlated on a range of spatial and temporal scales (Smith and Kohn, 2008). This correlated variability has a powerful influence on population coding and information processing capacity in neuronal networks, the extent and sign of which depends on its properties (Zohary et al., 1994; Shadlen and Newsome, 1998; Abbott and Dayan, 1999; Averbeck et al., 2006). Furthermore, a series of recent studies have shown that correlated variability can be modulated by instruction and experience (Cohen and Maunsell, 2009; Mitchell et al., 2009; Komiyama et al., 2010; Gu et al., 2011; Jeanne et al., 2013). Given the interaction between correlated variability and population coding, these findings suggest that spike count correlations might be indicative of the processing state of a particular population of neurons, or perhaps even actively modulated by the brain in order to improve processing in the relevant neuronal population. Unfortunately, correlated variability comes with a substantial limitation in that it can only be assayed over multiple repeated trials of an identical stimulus. In this report, we described how single-trial variance in normalized firing rate is inversely related to latent correlation, and we proposed that population variance might function as a single-trial proxy for spike count correlation.

Single-trial measures and trial-averaged measures have complementary strengths and weaknesses. While single-trial measures are typically noisier than their trial-averaged counterparts, they can reveal trial-to-trial dynamics of the phenomenon of interest. For example, an all-too-familiar aspect of attention performance is that it tends to lapse. However, studies of attention that use trial-averaged measures (such as mean reaction time, hit rate accuracy, or spike count correlations) implicitly assume that attention is deployed consistently across all the trials within a particular attention condition. While efforts such as these have certainly paid dividends, single-trial measures are needed to study within-condition fluctuations such as the dynamics of attentional deployment on a sub-second time scale. A measure of neuronal population response in terms of firing rate has recently been employed for a similar aim (Cohen and Maunsell, 2010, 2011). This is possible, of course, because attention modulates firing rates as well as correlated variability. Another example of the strengths of single-trial analysis is provided by the field of motor prosthetics, where both firing rate and higher-order statistics can be used to decode a motor action (Yu et al., 2009). We suggest that population variance can be used to study neuronal interactions in single-trial contexts, serving as a near instantaneous estimate of the latent correlation in a population in much the same way as spike count correlation has been used across trials. This measure could be employed in conjunction with firing rate or in isolation depending on the response characteristics of the population in the conditions under study.

Despite the good general correspondence between the *in vivo* data and the predictions of the simulations, the trend for our observed relationship between classification performance and Δ*r*_sc_ was slightly below that which was predicted (compare black dashed line to solid gray line in Figure 5). Several factors may contribute to this difference. Firstly, it may be that our simulated data did not match the actual neuronal data with respect to critical statistics that we did not explicitly control. We constructed our simulated data sets to match our *in vivo* data as closely as possible (e.g., number of neurons and average firing rate and correlation). Using the published algorithm of Macke and colleagues (2009), we were mostly successful in this aim, although an exact match on some of the desired parameters was not possible. Specifically, the variance in the pair-wise correlation within our simulated datasets tended to be much lower than what was observed in the real data, which may have affected classification performance. Effects carried by higher-order statistics of the *in vivo* data, such as skew or kurtosis, that we did not match with our simulations may have also affected classification. This does not present an inherent drawback, however, as future efforts may be able to leverage these higher moments for additional classification power, much as we have aimed to do here with population variance. Secondly, we implicitly assumed that latent correlation was constant for neurons located at a specific distance from each other. In actuality, and as we described in detail above, correlation is known to fluctuate due to a number of cognitive factors. Within-condition variance in latent correlation due to fluctuations in factors such as arousal, for example, would tend to reduce our classification performance compared to the simulation predictions. Nevertheless, our simulation findings match fairly well with the *in vivo* data (Figure 5), particularly given the restricted range of Δ*r*_sc_ values we observed, and this result strengthens our confidence in their predictive value.

For our simulation and *in vivo* data analyses we aimed to keep firing rate constant between conditions. We chose this in our simulations, and ensured it was true in the *in vivo* data by statistical comparison, to focus on the ability to classify correlation conditions on the basis of variance alone. Of course, most real world situations will present some differences in firing rate across conditions. It is important to take these firing rate differences into account because cross-trial variance in individual neurons tends to be directly related to firing rate due to their quasi-Poisson characteristics (Tolhurst et al., 1983). However, we should emphasize that cross-trial variance of individual neurons is theoretically an independent quantity from the variance in normalized firing rate across all neurons on a single trial (population variance). Intuitively, a single neuron might be highly variable or highly reliable, and that knowledge gives us no information about its covariance with the rest of the population. Thus, while the interaction between firing rate and the variance of individual neurons (Tolhurst et al., 1983) and the covariance of pairs of neurons (de la Rocha et al., 2007) have both been studied, it remains unclear how population variance scales with firing rate in real systems. This topic will require further research, and likely will require a multivariate approach to apply population variance as a measure of latent correlation in dynamic contexts. Single-trial approaches to parceling out the relative contributions of shared and independent variability to neuronal responses (Yu et al., 2009) may prove useful in disentangling cross-trial variance in individual neurons from population variance.

Our *in vivo* data were chosen and analyzed based on the previously demonstrated effect that distance affects correlation in V4 (Smith and Sommer, 2013). We used a resampling procedure to identify near and far pairs of neurons in the data, with the result that firing rates were well matched but correlation differed. While the differences in correlation that we observed were small (Δ*r*_sc_ ≈ 0.03; Figure 5), they were similar to what has been previously reported for correlation as a function of distance in V4 (Smith and Sommer, 2013). For these small differences in correlation we found a small but reliably better-than-chance classification performance (Figure 5). We also found that, as expected, greater differences in correlation lead to improved classification performance, and in a manner that closely matched the predictions of our computer simulations. If we consider other experimental contexts where larger differences in correlation have been reported, our computer simulations predict greatly improved classification performance. For example, Cohen and Maunsell (2011) found changes in correlation as large as Δ*r*_sc_ ≈ 0.1 in V4 during an attention task, while Gu and colleagues (2011) found changes in correlation as large as Δ*r*_sc_ ≈ 0.12 in area MST following a perceptual learning task. Our simulations predict that for effects of these magnitudes, classification performance approaching AUC = 0.7 would be achieved with around 120 neurons a quantity that is not unattainable even today, and which will likely be commonplace in the near future.

In this report we advanced the idea that population variance in normalized firing rate can be a useful tool for single-trial analyses. Since population variance is intimately related to correlation, variance itself is indicative of functional connectivity within a population of neurons. This is of paramount importance because the computational power of the brain comes from the interaction of neurons, and is best studied in the context of neuronal interactions. Single-trial measures are equally important because they enable us to study the dynamic fluctuations that inevitably characterize perceptual and cognitive processes as the brain interprets information from the natural world. We suggest that the use of variance as a fulcrum for understanding neuronal computations and measuring functional interactions is an essential development to make use of the massively parallel recordings that lie in our near future.

### Conflict of interest statement

The authors declare that the research was conducted in the absence of any commercial or financial relationships that could be construed as a potential conflict of interest.

## Appendix

### Mathematical proof of concept

Let **y** be an *n* × 1 random vector variable and let *E*[**y**] = **μ**, *Cov*[**y**] = **Σ**. In this context, each element *y*_*i*_ of **y** is a spike count from the *i*th of *n* neurons observed on a single trial. Each neuron *i* has a distribution of spike counts with mean μ_*i*_ and variance Σ_*ii*_. For any *n* × *n* symmetric matrix **A**, the product **y′Ay** is a *quadratic form* of **y**. It is known (Graybill, 1976, Chapter 4) that the expectation of this quadratic form is:

(1) $$E[{\text{y}}^{\prime }\text{Ay}]=tr\{\text{A}\Sigma \}-\mu^{\prime }\text{A}\mu$$

where *tr*{} denotes the trace operator. If we let $A=\frac{I-1(1/n)}{n-1}$, where **I** is an *n* × *n* identity matrix and **1** is an *n* × *n* matrix of ones, then the quadratic form **y′Ay** is the variance of **y**. In our framework, this quantity represents the single-trial variance across the population of neurons.

If we normalize the spike counts from each neuron by z-scoring, i.e., $z_{i}=\frac{y_{i}-\mu_{i}}{\sqrt{\Sigma_{ii}}}$, then **Σ** becomes equivalent to the correlation matrix, **C**. The expectation of the variance of **z** then becomes:

(2) $$E[{\text{z}}^{\prime }\text{Az}]=tr\{\text{AC}\}-0^{\prime }\text{A}0=tr\{\text{AC}\}$$

Next we will prove that the expectation of the variance of **z** equals one minus the average correlation between neurons.

A property of the trace operator is that the trace of the inner product of two matrices **AC** is equal to the sum of the Hadamard (or “element-wise”) product **A′** ◦ **C** (i.e., (**A′** ◦ **C**)_*i, j*_ = (**A′**)_*i,j*_ · (**C**)_*i, j*_). Since **A** is symmetric (by definition):

(3) $$tr\{\text{AC}\}=\sum_{i,j}{\left(\text{A}◦\text{C}\right)}_{i,j}$$

Hereafter, the subscripts *i* and *j* will be assumed. Substituting **A** as defined above:

(4a) $$tr\{\text{AC}\}=\sum \left(\left(\frac{1}{n-1}\left(\text{I}-\frac{1}{n}1\right)\right)◦\text{C}\right)$$

(4b) $$=\sum \left(\left(\frac{1}{n-1}\left(\frac{n-1}{n}\text{I}+\frac{1}{n}\text{I}-\frac{1}{n}1\right)\right)◦\text{C}\right)$$

(4c) $$=\sum \left(\left(\frac{1}{n}\text{I}+\frac{1}{n-1}\left(\frac{1}{n}\text{I}-\frac{1}{n}1\right)\right)◦\text{C}\right)$$

(4d) $$=\sum \left(\frac{1}{n}\text{I}◦\text{C}+\frac{1}{n(n-1)}\left(\text{I}-1\right)◦\text{C}\right)$$

(4e) $$=\sum \left(\frac{1}{n}\text{I}◦\text{C}\right)+\sum \left(\frac{1}{n(n-1)}\left(\text{I}-1\right)◦\text{C}\right)$$

Since **C** is a correlation matrix, all diagonal elements are equal to 1, meaning **I** ◦ **C** = **I**. Further still, trivially, $\sum \left(\frac{1}{n}I\circ C\right)=\sum \left(\frac{1}{n}I\right)=1$. It follows that:

(5) $$tr\{\text{AC}\}=1-\frac{1}{n(n-1)}\sum \left(\left(1-\text{I}\right)◦\text{C}\right)$$

The term containing the sum is the average of all of the off-diagonal elements of **C**; that is, the average pairwise correlation between the population of neurons. From (2), we have that the expectation of the single-trial variance across the neuron population is equal to *tr*{**AC**}:

(6) $$E[{\text{z}}^{\prime }\text{Az}]=tr\{\text{AC}\}=1-\frac{1}{n(n-1)}\sum \left(\left(1-\text{I}\right)◦\text{C}\right)\;□$$

Thus, the expectation of the single-trial variance in normalized firing rate across a population of neurons is precisely equal to one minus the average pairwise correlation between neurons in that population.

This relationship is a generalization of the well-known Law of Total Variance, which explains that the total variance in a random variable *Y*, when conditioned on some random variable *X*, is equal to sum of the expectation of the variance and the variance of the expectation. That is, *Var*[*Y*] = *E*[*Var*[*Y*|*X*]] + *Var*[*E*[*Y*|*X*]] (Weiss et al., 2005). These two terms are also referred to as the “unexplained variance” and “explained variance” (the variance of *Y* that can be explained by *X*), respectively. In our case, we have *n* random variables (neurons), each of which is explained in some part by the other *n* − 1 variables in accordance with the correlation matrix. As mentioned above, **y′Ay** is the variance on a single trial, and *E*[**y′Ay**] is the expectation of that variance (eq. 1). Since μ is the expectation of **y**, **μ′A****μ** represents the variance of the expectation. Finally, *tr*{**AΣ**} represents the total variance of the random vector variable **y**.

Although we have proved here that the expectation of the single-trial variance is the complement to correlation, we have assumed that the true means (**μ**) and variances (*diag*(**Σ**)) of the spike counts (**y**) must be known in order to standardize the variables. In practice, however, these statistics can only be estimated, and this limitation will introduce error to the estimate of correlation based on the measurement of single-trial variance. This is the typical problem of sampling error, and it can be mitigated by sampling a greater number of observations of spike counts to better estimate the statistics of the spike count distributions of the neurons under observation.
